# MixedBayes: An R Package for Longitudinal Gene–Environment Interaction Analysis Using Robust Sparse Bayesian Mixed Models

**DOI:** 10.3390/e28060649

**Published:** 2026-06-09

**Authors:** Kun Fan, Xiaoxi Li, Shejuty Devnath, Brock Olson, Cen Wu

**Affiliations:** 1 Department of Biostatistics and Data Science, The University of Texas Health Science Center at Houston School of Public Health, Houston, TX 77030, USA; kun.fan@uth.tmc.edu; 2 Department of Statistics, Kansas State University, Manhattan, KS 66506, USA; xiaoxili@ksu.edu (X.L.); shejuty@ksu.edu (S.D.); brock.olson@yahoo.com (B.O.)

**Keywords:** quantile mixed-effects model, robust Bayesian variable selection, high-dimensional statistical inference, repeated measures, uncertainty quantification

## Abstract

Robust variable selection methods have emerged as powerful tools for dissecting high-dimensional gene–environment interactions in longitudinal studies, owing to their ability to accommodate intra-cluster correlations, capture structured sparsity, and handle heavy-tailed repeated measures. Despite these advantages, variable selection-based interaction analysis still suffers from a lack of valid inferential tools to quantify the uncertainty associated with important gene–environment interactions. In this paper, we introduce the R package mixedBayes (version 0.2.5), which implements fully Bayesian robust mixed-effects models proposed in recent work for high-dimensional longitudinal gene–environment interaction analysis. Specifically, the package considers two major classes of mixed models. The first accommodates interactions between omics features and treatment effects arising from repeated-measures one-way ANOVA with high-dimensional genetic factors. The second provides a more general framework for modeling interactions between individual genetic main effects and environmental factors. Both models enable posterior Bayesian inference via Markov chain Monte Carlo (MCMC). We provide detailed numerical examples and accompanying R code to facilitate robust interaction analysis using mixedBayes. In addition, a case study based on longitudinal asthma data with high-dimensional SNP measurements is presented.

## 1. Introduction

Variable selection and hypothesis testing are the two primary statistical frameworks for conducting gene–environment (G×E) interaction analysis [1,2,3]. Due to the high dimensionality of genetic factors, G×E analysis is well-suited to penalized variable selection methods. In particular, since outlying disease outcomes are frequently encountered owing to heterogeneity of complex diseases, robust variable selection methods have been developed for G×E interaction studies [4,5,6,7]. Despite these technical advances, a major limitation of penalized interaction analysis is the lack of uncertainty quantification. In contrast to statistical test-based methods that routinely provide p-values, regularized variable selection and its extensions in gene–environment interaction studies struggle to deliver valid inferential measures. This issue is further exacerbated in robust variable selection, where non-differentiable loss functions make it even more challenging to establish the asymptotic properties and resulting inference procedures in terms of confidence intervals, p-values, and false discovery rates (FDRs).

Longitudinal G×E analysis poses an additional layer of challenge for high-dimensional inference, as both phenotypic responses and omics predictors are repeatedly measured over time [1]. Consequently, intra-cluster correlations must be appropriately modeled. Statistical inference for high-dimensional longitudinal data has been addressed in only a limited number of frequentist studies, including penalized generalized estimating equations (PGEEs) [8] and high-dimensional mixed-effects models developed using de-biased LASSO [9,10]. Inference procedures of these studies are valid only under Gaussian errors, as GEE is known to be sensitive to even a single outlier [11], and the de-biased LASSO [12,13] has been shown to exhibit inferior performance in variable selection and statistical inference under data heterogeneity in empirical studies [14]. Robust inference, therefore, remains an important yet largely underexplored problem for frequentist longitudinal interaction studies.

Robust, fully Bayesian methods have recently emerged as a promising approach for inference under skewed model errors in longitudinal interaction studies. Among existing studies, Fan et al. (2025) [15] have developed a Bayesian quantile mixed-effects model for longitudinal treatment-by-genetic interaction analysis under a repeated-measures one-way ANOVA framework, where interactions are modeled at the group level between treatment indicators and lipid features. In addition, Li et al. (2025) [16] have considered a more general interaction setting, where interactions are modeled at the individual level as products between environmental factors and high-dimensional genetic variables. Both models and the alternatives are implemented in the R package mixedBayes, which is available on CRAN.

To better understand the advantages and limitations of the mixedBayes package for analyzing repeated-measures data, we provide a non-exhaustive list of R packages for sparse longitudinal data analysis in Table 1, which covers diverse methodological frameworks such as mixed-effects models, generalized estimation equations (GEEs) [17], fully Bayesian analysis, and tree-based machine learning, among others. While all these tools are well developed for repeated-measures data, only a small subset—namely, springer [18,19], interep [20,21], and mixedBayes [15]—are tailored for high-dimensional longitudinal interaction studies. Among them, springer achieves robustness through the quadratic inference function (QIF) framework [22], whereas mixedBayes attains robustness via a robust likelihood formulation.

Statistical inference in such settings remains particularly challenging. Among the listed packages, only PGEE, plsmmLasso and mixedBayes provide high-dimensional inference procedures, with mixedBayes further offering robust inference in the presence of outliers in phenotypic responses. It is also worth noting that mixed-effects models can be extended to incorporate tree-based methods, such as decision trees and random forests, for analyzing longitudinal data [23]. Packages such as MultivariateRandomForest and LongituRF can perform variable selection via variable importance measures. However, tree-based machine learning typically prioritizes predictive performance over statistical inference and, thus, lacks principled uncertainty quantification measures.

Overall, Table 1 highlights a notable gap in current tools. The mixedBayes package uniquely addresses this gap by integrating high-dimensional longitudinal modeling, interaction analysis, and robust statistical inference within a unified Bayesian framework. In this paper, we focus on the practical implementation of the Bayesian longitudinal analysis frameworks proposed in Fan et al. (2025) [15] and Li et al. (2025) [16], implemented via the R package mixedBayes. Spike-and-slab priors are incorporated in both robust Bayesian mixed models and play a critical role in conducting variable selection and posterior inference. Specifically, Fan et al. (2025) [15] adopt the median probability model (MPM) [24] to identify individual-level main effects and group-level G×E interactions, whereas Li et al. (2025) [16] perform the ranking and selection of important main and interaction effects using a Bayesian false discovery rate (FDR)-assisted procedure based on posterior inclusion probabilities (PIPs) derived from spike-and-slab priors. We illustrate the use of the mixedBayes package through detailed instructions and numerical examples, demonstrating how to implement median probability models (MPMs) and Bayesian FDR-based procedures. The R package mixedBayes (version 0.2.5) is available at https://cran.r-project.org/package=mixedBayes (accessed on 23 April 2026).

**Table 1:** An incomplete list of R packages for sparse longitudinal data analysis in moderate- and/or high-dimensional settings.

Package	Method	Application	Robust	Inference	References
mixedBayes	Bayesian	G×E interaction	Yes	Yes	[15,16]
bayeslongitudinal	Bayesian	Main effect modeling	No	Yes	[25]
geeVerse	GEE	Main effect modeling	Partially	No	[26]
springer	QIF [22]	G×E interaction	Yes	No	[18,19]
interep	GEE	G×E interaction	No	No	[20,21]
PGEE	GEE	Main effect modeling	No	Yes	[8,27]
pgee.mixed	GEE	Medical Expenditure Panel Survey (MEPS)	No	Yes	[28]
OmicPred	Mixed model	Alzheimer’s Disease Neuroimaging Initiative (ADNI)	No	No	[29]
plsmmLasso	Mixed model	Oral Microbiome in Early Infancy (OMEI) study	No	Yes	[10]
glmmLASSO	GLMM	Longitudinal and clustered data	No	No	[30]
REEMtree	Tree-based mixed model	Transaction data and accident fatality data	No	No	[31]
Multivariate- RandomForest	Tree-based	Multivariate outcome prediction	No	No	[32,33]
LongDat	GLMM	longitudinal microbiome studies	No	Yes	[34]
LongituRF	Tree-based mixed model	HIV vaccine trial with 20,000 gene transcripts	No	No	[35,36]

## 2. Two Major Longitudinal Interaction Models

Before we introduce the two major robust Bayesian longitudinal interaction models implemented in the R package mixedBayes [15,16], let us first review the prototype G×E model in non-robust, low-dimensional settings. A standard gene–environment interaction model with the environmental main effect $E_{i}$, and genetic main effect $G_{i}$, as well as their interaction $E_{i}G_{i}$ under continuous disease phenotype $Y_{i}$ for the *i*th subject (i=1,...,n), can be expressed as

(1) $$E\left(Y_{i}\right)=\beta_{0}+\beta_{1}E_{i}+\beta_{2}G_{i}+\beta_{3}E_{i}G_{i},$$

where $\beta_{1}$, $\beta_{2}$ and $\beta_{3}$ are regression coefficients representing main environmental, genetic and interaction effects. In frequentist G×E studies, uncertainty quantification can be performed by testing the significance of these coefficients. In a one-way ANOVA setting with (for example) 3 treatment levels denoted using the environmental factor $E_{i}$, the baseline model (1) leads to

$$E\left(Y_{i}\right)=\beta_{0}+\beta_{1}E_{1i}+\beta_{2}E_{2i}+\beta_{3}G_{i}+\left(\beta_{4}E_{1i}+\beta_{5}E_{2i}\right)G_{i},$$

where $E_{1i}$ and $E_{2i}$ are binary indicators representing the categorical E factor $E_{i}$ for the *i*th subject. Statistical inference becomes increasingly challenging when the disease phenotype is repeatedly measured in the presence of outliers, as the genetic factors are high-dimensional and G×E interactions are of a group structure, motivating the development of sparse Bayesian quantile mixed-effects models implemented in the R package mixedBayes [15]. Moreover, under heavy-tailed model errors, Li et al. (2025) [16] have considered a direct extension of model (1) to a high-dimensional case without requiring a grouping structure of environmental factors. Next, we present the two longitudinal models in more detail.

### 2.1. Bi-Level Sparse Bayesian Quantile Mixed-Effects Model

In a longitudinal study with *n* subjects observed at *k* time points, let $Y_{it}$ denote the phenotype and $G_{\mathrm{it}}$∈$R^{p}$ and $E_{\mathrm{it}}$∈$R^{q}$ denote the genetic and categorical treatment factors for subject *i* at time *t* (1≤i≤n,1≤t≤k) such that $G_{\mathrm{it}}={\left(G_{it1},\ldots ,G_{itp}\right)}^{⊤}$ and $E_{\mathrm{it}}={\left(E_{it1},\ldots ,E_{itq}\right)}^{⊤}$, respectively. The time-dependent $E_{\mathrm{it}}$ includes classical repeated-measures ANOVA with time-invariant factors as a special case. To model main and interaction effects under longitudinal phenotypes with skewed distributions, Fan et al. (2025) [15] consider the following bi-level quantile mixed-effects model at a specified quantile level θ(0<θ<1),

(2) $$Y_{it}={T_{\mathrm{it}}}^{⊤}\beta_{0,\theta }+{E_{\mathrm{it}}}^{⊤}\beta_{1,\theta }+{G_{\mathrm{it}}}^{⊤}\beta_{2,\theta }+{\left(G_{\mathrm{it}}⨂E_{\mathrm{it}}\right)}^{⊤}\beta_{3,\theta }+{Z_{\mathrm{it}}}^{⊤}\alpha_{i,\theta }+\epsilon_{it,\theta }.$$

where “bilevel” refers to modeling main effects at the individual level and interaction effects at the group level. The model includes fixed effects $\beta_{0,\theta }$, $\beta_{1,\theta }$, $\beta_{2,\theta }$, and $\beta_{3,\theta }$, corresponding to time effects $T_{\mathrm{it}}$ (including an intercept term), treatment factor $E_{\mathrm{it}}$, genetic factor $G_{\mathrm{it}}$, and treatment – genetic interactions $G_{\mathrm{it}}⊗E_{\mathrm{it}}$, respectively. The interaction term $G_{\mathrm{it}}⊗E_{\mathrm{it}}$ is a pq-dimensional Kronecker product:

$$G_{\mathrm{it}}⊗E_{\mathrm{it}}={\left[G_{it1}E_{it1},\ldots ,G_{itp}E_{itq}\right]}^{⊤}.$$

Model (2) induces correlations among repeated measures through subject-specific random effects $\alpha_{i,\theta }\in R^{h}$. The time covariate $Z_{\mathrm{it}}\in R^{h}$ represents a random intercept model when h=1, and a random intercept–slope model when h=2. The model errors $\epsilon_{it,\theta }$ are assumed to be independent, with their θth quantile being zero. For notational simplicity, we omit the subscript “θ” on the fixed and random effects hereafter.

**Robust likelihood.** We refer readers to Fan et al. (2025) [15] for details on specifying the robust likelihood for model (2) based on the asymmetric Laplace distribution (ALD) [37,38,39], which has been adopted in Bayesian regularized quantile regressions that yield valid inference measures in high-dimensions [14]. We acknowledge that several other robust likelihood functions have been studied in sparse linear regression models, but have not yet been explored under mixed-effects models [40,41].

**Bi-level shrinkage priors.** To identify important main and interaction effects, we assign structured shrinkage priors to the fixed-effect coefficients $\beta_{1}$ = ${\left(\beta_{11},\ldots ,\beta_{1q}\right)}^{⊤}$, $\beta_{2}$ = ${\left(\beta_{21},\ldots ,\beta_{2p}\right)}^{⊤}$, and $\beta_{3}={\left(\beta_{31},\ldots ,\beta_{3p}\right)}^{⊤}$, where $\beta_{3g}$ = ${\left(\beta_{3g1},\ldots ,\beta_{3gq}\right)}^{⊤}$ for g=1,…,p. These correspond to environmental main effects, genetic main effects, and their interactions, respectively.

To select group-level interaction effects, we assign the following multivariate spike-and-slab priors,

$$\begin{array}{cc} \beta_{3g}|\phi_{2g},s_{2g} & ∼\phi_{2g}N_{q}\left(0,s_{2g}I_{q}\right)+\left(1-\phi_{2g}\right)\delta_{0}\left(\beta_{3g}\right),\\ \phi_{2g} & ∼\mathrm{Bernoulli}(\pi_{2}),\\ s_{2g} & ∼\mathrm{Gamma}\left(\frac{q+1}{2},\frac{\eta_{2}^{2}}{2}\right), \end{array}$$

where $\delta_{0}\left(\cdot \right)$ denotes a point mass at zero. The binary latent variable $\phi_{2g}$ indicates whether the interaction effect for the *g*th genetic factor is selected ($\phi_{2g}=1$) or excluded ($\phi_{2g}=0$). When $\phi_{2g}=1$, the hierarchical prior reduces to a multivariate Laplace prior, corresponding to the Bayesian quantile group LASSO. By integrating out $\phi_{2g}$ and $s_{2g}$, we obtain the following multivariate spike-and-slab prior,

$$\beta_{3g}|\eta_{2}∼\pi_{2}M\text{-}\mathrm{Laplace}\left(\beta_{3g}|\eta_{2}\right)+\left(1-\pi_{2}\right)\delta_{0}\left(\beta_{3g}\right),$$

where $\pi_{2}\in \left[0,1\right]$.

To identify individual-level genetic main effects, we impose univariate spike-and-slab priors,

$$\begin{array}{cc} \beta_{2g}|\phi_{1g},s_{1g} & ∼\phi_{1g}N\left(0,s_{1g}\right)+\left(1-\phi_{1g}\right)\delta_{0}\left(\beta_{2g}\right),\\ \phi_{1g} & ∼\mathrm{Bernoulli}(\pi_{1}),\\ s_{1g} & ∼\mathrm{Gamma}\left(1,\frac{\eta_{1}^{2}}{2}\right), \end{array}$$

where the parameter $\pi_{1}\in \left[0,1\right]$. When $\phi_{1g}=1$, this prior reduces to a Laplace shrinkage prior; otherwise, $\beta_{2g}=0$, indicating no main effect for gene *g*.

### 2.2. FDR-Assisted Robust Sparse Bayesian Linear Mixed Model

Li et al. (2025) [16] have considered a more general extension of model (1) in a longitudinal interaction study with *n* subjects and *k* repeated measures for each observation. For the *i*th subject (1≤i≤n), the disease trait $Y_{it}$, genetic factors $G_{\mathrm{it}}$ = ${\left(G_{it1},\ldots ,G_{itp}\right)}^{⊤}$, and environmental factors $E_{\mathrm{it}}$ = ${\left(E_{it1},\ldots ,E_{itq}\right)}^{⊤}$ are measured at time point *t* (1≤t≤k). Li et al. (2025) [16] have proposed the following robust longitudinal mixed-effects model,

(3) $$Y_{it}={X_{\mathrm{it}}}^{⊤}\gamma_{0}+{E_{\mathrm{it}}}^{⊤}\gamma_{1}+{G_{\mathrm{it}}}^{⊤}\gamma_{2}+{\left(G_{\mathrm{it}}⨂E_{\mathrm{it}}\right)}^{⊤}\gamma_{3}+{Z_{\mathrm{it}}}^{⊤}\alpha_{i}+\epsilon_{it},$$

where $X_{\mathrm{it}}$ represents the time effects (including an intercept term). To distinguish from the bi-level quantile mixed model in Section 2.1, we use γ to denote fixed effects in mixed model (3), with the order of genetic and environmental main effects adjusted to match variable dimensions. The fixed effects $\gamma_{1}$∈$R^{q}$, $\gamma_{2}$∈$R^{p}$, and $\gamma_{3}$∈$R^{pq}$ correspond to the genetic main effects, environmental main effects, and their interactions, respectively. The interaction effects are constructed via the Kronecker product:

$$G_{\mathrm{it}}⨂E_{\mathrm{it}}={\left[G_{it1}E_{it1},G_{it1}E_{it2},\ldots ,G_{it1}E_{itq},G_{it2}E_{it1},\ldots ,G_{itp}E_{itq}\right]}^{⊤}.$$

In model (3), the random effect $\alpha_{i}$ and associated time effects $Z_{\mathrm{it}}$ are defined the same as in model (2). For a random intercept–slope model, $Z_{\mathrm{it}}$ = ${\left(1,t\right)}^{⊤}$ and $\alpha_{i}$∈$R^{2}$; for a random intercept model, $Z_{\mathrm{it}}$ = 1 and $\alpha_{i}$ reduces to a scalar. The error terms $\epsilon_{it}$ are assumed to be independent and follow a heavy-tailed distribution to ensure robustness against outliers.

Model (3) differs from model (2) in that it is not derived from a repeated-measures one-way ANOVA framework; thus, the environmental factors are not restricted to categorical variables. Consequently, the group-level interaction structure in model (2) no longer exists, and all main and interaction effects are modeled at the individual level.

**Robust likelihood and shrinkage priors**

Li et al. (2025) [16] have adopted a Laplace distribution to build the robust likelihood function. The following shrinkage priors are adopted to detect important main and interaction effects.

For genetic main effects, we assign univariate spike-and-slab priors,

$$\begin{array}{cc} \gamma_{2g}|\phi_{1g},s_{1g} & ∼\phi_{1g}N\left(0,s_{1g}\right)+\left(1-\phi_{1g}\right)\delta_{0}\left(\gamma_{2g}\right),\\ \phi_{1g} & ∼\mathrm{Bernoulli}(\pi_{1}),\\ s_{1g} & ∼\mathrm{Gamma}\left(1,\frac{\eta_{1}^{2}}{2}\right). \end{array}$$

where the parameter $\pi_{1}\in \left[0,1\right]$ and g=1,…,p. For interaction effects, we also impose univariate spike-and-slab priors,

$$\begin{array}{cc} \gamma_{3l}|\phi_{2l},s_{2l} & ∼\phi_{2l}N\left(0,s_{2l}\right)+\left(1-\phi_{2l}\right)\delta_{0}\left(\gamma_{3l}\right),\\ \phi_{2l} & ∼\mathrm{Bernoulli}(\pi_{2}),\\ s_{2l} & ∼\mathrm{Gamma}\left(1,\frac{\eta_{2}^{2}}{2}\right). \end{array}$$

where l=1,…,pq and the parameter $\pi_{2}\in \left[0,1\right]$.

**Remark** **1.** *The difference between model (2) and model (3) is that model (2) is designed for a repeated-measures one-way ANOVA setting, where the environmental factors correspond to groups of binary treatment indicators, and the associated gene–environment interactions, therefore, possess a grouped structure. In contrast, model (3) does not impose such a grouping restriction, and the gene–environment interactions are modeled individually rather than at the group level. Therefore, model (3) is not applicable to one-way ANOVA setting since it violates the grouping structure. In addition, model (3) also differs from model (2) in its variable selection criterion. Specifically, model (2) employs the median probability model (MPM) [24,42], using a cutoff of 0.5 for posterior inclusion probabilities to determine selected predictors. In contrast, Li et al. (2025) [16] propose using a Bayesian FDR-controlled adaptive threshold as the cutoff for variable selection in model (3). We also note that, since the environmental main effects are not subject to selection under both models, the resulting specifications satisfy the weak hierarchy in interaction studies [6,43].*

### 2.3. Additional Priors

For both models (2) and (3), described in Section 2.1 and Section 2.2, respectively, we assign normal priors to the subject-specific random effects. Under a random intercept–slope model, $\alpha_{i}∼N\left(0,\phi^{2}I\right)$, and under a random intercept-only model, $\alpha_{i}$$∼N(0,\phi^{2})$.

We specify Beta hyperpriors for the inclusion probabilities, $\pi_{1}∼\mathrm{Beta}\left(r_{1},w_{1}\right)$ and $\pi_{2}∼\mathrm{Beta}\left(r_{2},w_{2}\right)$, and Gamma priors for the shrinkage parameters, $\eta_{1}^{2}∼\mathrm{Gamma}\left(a_{1},b_{1}\right)$, $\eta_{2}^{2}∼\mathrm{Gamma}\left(a_{2},b_{2}\right)$, and τ∼Gamma(c,d). For the variance of the random effects, we impose an inverse-gamma prior, $\phi^{2}∼\mathrm{IG}\left(e,f\right)$. Unless otherwise specified, we set these hyperparameters to 1 to reflect diffuse prior choices. A detailed hyperparameter sensitivity analysis can be found in Section B.6 of the Appendix in Fan et al. (2025) [15], which suggests that the model performance is not sensitive to the choice of hyperparameters. Therefore, for convenience of use, the current *mixedBayes* package sets all hyperparameters to 1 by default. Please refer to Fan et al. (2025) [15] and Li et al. (2025) [16] for derivations of the Gibbs samplers and associated MCMC algorithms.

## 3. R Package mixedBayes

The mixedBayes package provides a unified framework for fitting Bayesian regularized quantile mixed models in longitudinal gene–environment (G×E) interaction studies. The core function, mixedBayes(), implements robust sparse mixed-effect modeling proposed for the aforementioned two major longitudinal interaction studies [15,16]. The package accommodates bi-level sparsity through structured spike-and-slab priors [15] and supports robust quantile modeling based on the asymmetric Laplace distribution. Alternative methods using non-sparse Laplace priors and/or non-robust Gaussian likelihoods are also included. To ensure computational efficiency, the MCMC algorithms are implemented in C++. In addition to the core modeling function, the package includes several supporting utilities, such as reformat(), predict_mixedBayes(), and selection(), which facilitate data pre-processing, model-based prediction, and variable selection, respectively.

### 3.1. Data Pre-Processing Utilities

The package requires all input data (response, genetic, and environmental factors) to be in the long format. It provides one auxiliary function, reformat(), to prepare long-format longitudinal data. The mixedBayes() function automatically constructs gene–environment (G×E) interaction terms internally using the Kronecker product of the genetic and environmental factors, as described in Section 2.1 and Section 2.2. Therefore, users only need to provide the main-effect design matrices, without explicitly specifying the interaction matrix. The function reformat() converts repeated-measures data into the required long format. For example, when the response is stored in wide format (subjects in rows and time points in columns), reformat() transforms it into a stacked vector indexed by subject and time. It also expands subject-level covariates to align with the longitudinal design.

As an illustration, consider a study with 200 subjects, each with the response variable measured at five time points, along with 100 genetic factors and three environmental factors. The cross-sectional genetic and environmental matrices have dimensions of 200×100 and 200×3, respectively. The function reformat(5, y, type = “r”) expands the phenotypic response to a 1000×1 vector, while reformat(5, g, type = “d”) and reformat(5, e, type = “d”) expand the genetic and environmental matrices to dimensions of 1000×100 and 1000×3, respectively, thereby matching the long-format structure. The current version of mixedBayes() assumes a longitudinal response, with genetic and environmental factors treated as cross-sectional covariates.

#### The Core Functions

In the R package mixedBayes, the core function is mixedBayes(y, e, X, g, k, iterations, burn.in, slope, robust, quant, sparse, structure). It fits a Bayesian longitudinal regularized quantile mixed model tailored for high-dimensional gene–environment interaction (G×E) studies under repeated measurements. The input arguments y, e, X, and g correspond to the response vector, environmental covariates (or treatment indicator groups), time-effect covariates (including an intercept term), and genetic factors, respectively. The argument k denotes the number of repeated measurements per subject. The argument slope specifies the type of mixed-effects models: if slope = TRUE, a random intercept-and-slope model is used; otherwise, a random intercept model is fitted. The robust option enables quantile modeling using an asymmetric Laplace distribution, with the quantile level specified by quant. When robust = FALSE, the model uses a Gaussian likelihood function. Sparsity structures can be imposed via the arguments structure and sparse. When sparse = TRUE, spike-and-slab priors are adopted to induce exact sparsity. If sparse = FALSE, Laplacian shrinkage priors are used instead, and exact sparsity cannot be achieved. When structure=“bilevel”, bi-level selection on main and interaction effects will be conducted corresponding to individual and group levels, respectively, as described in Fan et al. (2025) [15]. When structure=“individual”, selections only on individual-level G and E effects will be performed, as shown in Li et al. (2025) [16].

The function returns a mixedBayes object containing posterior summaries, including posterior median estimates of the model parameters, which are used for subsequent performance evaluation. A total of 2 (robust vs. non-robust) × 2 (random intercept vs. intercept-and-slope) × 2 (bi-level vs. individual structure) × 2 (spike-and-slab vs. Laplacian) =16 models are available. The number of MCMC iterations and the burn-in period are specified through arguments iterations and burn.in, respectively. If burn.in is set to NULL, no burn-in is applied, and posterior samples from all MCMC iterations are retained for estimation and inference.

### 3.2. Bayesian Variable Selection

In Bayesian hierarchical models, shrinkage priors determine how variable selection is conducted. The selection() function in the package mixedBayes implements two criteria: the median probability model (MPM) for spike-and-slab methods and 95% credible intervals for Laplace prior-based methods. In addition, the Bayesian FDR approach can be applied to spike-and-slab models to select important features under FDR control. Further details are provided below.

#### 3.2.1. Median Probability Model (MPM) Criterion

The treatment variable considered in the repeated-measure one-way ANOVA setting in Fan et al. (2025) [15] is categorical, leading to a group of binary indicators (dummy variables). By setting structure=“bilevel", the package performs variable selection on the main effects at the individual level and the interaction effects at the group level simultaneously.

Variable selection on the returned mixedBayes object is performed using function selection(), which extracts relevant main and interaction effects based on posterior samples generated from MCMC. When sparse = TRUE, selection follows the median probability model (MPM) strategy [24,42]. Let *S* denote the number of retained MCMC iterations after discarding burn-ins, and let $\delta_{j}^{\left(s\right)}\in \left\{0,1\right\}$ indicate whether the *j*th predictor is included in the model at iteration *s*. Then, the posterior inclusion probability (PIP) for the *j*th predictor is computed as

$$\pi_{j}=\frac{1}{S}\sum_{s=1}^{S}\delta_{j}^{\left(s\right)},\;j=1,\ldots ,d,$$

where *d* represents the total number of main and interaction effects subject to selection. Under the MPM criterion, predictors with $\pi_{j}\geq 0.5$ stay in the final model, yielding a sparse representation that keeps signals with strong posterior support while excluding irrelevant ones. The function selection() returns a binary indicator vector, where each component indicates whether the corresponding feature (main or interaction effect) is selected (denoted as 1) or not (denoted as 0).

#### 3.2.2. 95% Credible Interval Criterion

Among the 16 models implemented in the R package mixedBayes, 8 are based on Laplace shrinkage, for which the MPM is not applicable. In this case, setting sparse = FALSE enables variable selection based on posterior credible intervals: a predictor is selected if the corresponding 95% credible interval for its coefficient does not contain zero. The output of the selection() function is also a binary indicator vector specifying whether each effect is selected.

#### 3.2.3. Bayesian False Discovery Rate (FDR) Criterion

Under spike-and-slab priors, posterior inclusion probabilities (PIPs) quantify the strength of association between each main effect or interaction effect and the longitudinal response. Accordingly, PIPs can be used to rank their importance. In contrast to the fixed 0.5 cutoff in the MPM criterion, Bayesian FDR procedures yield adaptive thresholds that may improve performance in certain settings. Methodological developments on using Bayesian FDR to construct adaptive selection thresholds, as well as applications in genomics studies, are available in [44,45,46], among others. By construction, such adaptive thresholds are largely model-independent in the sense that, once spike-and-slab priors are incorporated into a Bayesian hierarchical model, the thresholds can be readily computed based on the posterior properties induced by the spike-and-slab formulation. This approach has demonstrated good empirical performance in main effect models [44,46]. Li et al. (2025) [16] have further considered it in gene–environment interaction studies by proposing a Bayesian FDR approach for selecting important main and interaction effects in robust linear mixed models for longitudinal interaction studies, which can be conducted using the package mixedBayes. Li et al. (2025) [16] have also demonstrated through numerical studies that, under repeated data generation from the true underlying model, the corresponding Bayesian FDR procedure effectively controls the overall proportion of false discoveries near the nominal level, supporting its practical utility in interaction models.

Specifically, when structure=“individual”, variable selection is performed at the individual predictor level for both main and interaction effects [16]. Variable selection performance is evaluated using the Bayesian false discovery rate (FDR) defined in Equation (4) below. For a given cutoff τ∈(0,1), all effects satisfying $\pi_{j}>\tau$ are declared as discoveries. The corresponding Bayesian FDR is defined as

(4) $$\mathrm{FDR}\left(\tau \right)=\frac{\sum_{j=1}^{d}\left(1-\pi_{j}\right)\;I\left(\pi_{j}>\tau \right)}{\sum_{j=1}^{d}I\left(\pi_{j}>\tau \right)},$$

where $1-\pi_{j}$ represents the posterior probability that the *j*th effect is null and I(·) is the indicator function. This quantity can be interpreted as the expected proportion of false discoveries among all selected main and interaction effects. To identify important effects under FDR control, we first sort the PIPs in descending order,

$$\pi_{\left(1\right)}\geq \pi_{\left(2\right)}\geq \cdots \geq \pi_{\left(d\right)}.$$

If the top *m* predictors are selected (m=1,...,d), the corresponding Bayesian FDR can be written as

$$\mathrm{FDR}\;\left(\pi_{\left(m\right)}\right)=1-\frac{1}{m}\sum_{i=1}^{m}\pi_{\left(i\right)}.$$

Given a target FDR level c∈(0,1), we choose

$$m^{*}=\max\left\{m:\mathrm{FDR}\;\left(\pi_{\left(m\right)}\right)\leq c\right\},\;\hat{\tau }=\pi_{\left(m^{*}\right)}.$$

All predictors with $\pi_{j}>\hat{\tau }$ are then selected. This adaptive strategy determines the selection threshold directly from the posterior evidence and guarantees that the expected proportion of false discoveries among the selected predictors is controlled at the prespecified level *c*. We refer readers to the numerical studies in Li et al. (2025) [16], which demonstrate that the Bayesian FDR is well controlled while achieving satisfactory identification performance in the presence of heavy-tailed repeated measurements.

### 3.3. Prediction Function

In addition to parameter estimation and variable selection, the package mixedBayes also provides functionality for model-based prediction. Given a fitted mixedBayes object, predictions for repeated-measurement outcomes can be obtained using the predict_mixedBayes() function. This function computes fitted values using posterior median estimates of the fixed effects—corresponding to time-effect covariates, environmental effects, and genetic main effects—as well as subject-specific random effects. Gene–environment interaction terms are automatically constructed internally and incorporated into prediction and, therefore, do not need to be supplied by the user. Depending on the specification of the logical argument slope, the function accommodates either a random intercept model or a random intercept-and-slope model. Predicted responses are obtained using posterior median estimates of both fixed and random effects, and prediction accuracy is summarized by the mean absolute error (MAE) or mean squared error (MSE) between the observed and predicted outcomes. Specifically, let ${\hat{y}}_{it}$ denote the corresponding fitted value computed using the posterior median. Then, MAE is defined as

$$\mathrm{MAE}=\frac{1}{nk}\sum_{i=1}^{n}\sum_{t=1}^{k}\left|y_{it}-{\hat{y}}_{it}\right|,$$

and MSE is defined as

$$\mathrm{MSE}=\frac{1}{nk}\sum_{i=1}^{n}\sum_{t=1}^{k}{\left(y_{it}-{\hat{y}}_{it}\right)}^{2},$$

The function returns an object of class mixedBayes.pred, which contains the vector of predicted response values for the repeated measurements, as well as the associated prediction error.

## 4. Simulation Examples

We illustrate how to use the R package mixedBayes to perform high-dimensional longitudinal interaction analyses [15,16]. Consider the random intercept-and-slope models (2) and (3) described in Section 2.1 and Section 2.2, respectively. Let *n* denote the sample size, *p* the number of genetic factors, *q* the number of environmental factors, and *k* the number of repeated measurements. Under both models, we generate a dataset with n=200 subjects, p=100 genetic factors, q=3 environmental factors, and k=5 time points per subject. The generation of environmental factors differs between models (2) and (3); see Section 4.1 and Section 4.2 for details. For both models, the genetic factors g are simulated from a multivariate normal distribution with an AR(1) covariance structure, marginal variance 1, and autocorrelation coefficient ρ=0.5. Detailed R codes for data generation and analysis using the mixedBayes package are provided below.

### 4.1. Bi-Level Modeling Under Bayesian Regularized Quantile Mixed Model

#### 4.1.1. Variable Selection Using the MPM

In the repeated-measure one-way ANOVA design with high-dimensional genetic factors introduced in Section 2.1, the environmental factors are a group of dummy variables indicating treatment groups. Gene–environment interactions, denoted by w, are then constructed internally in the package mixedBayes as the Kronecker product of the genetic and environmental factors. In the data-generating codes provided below, the coefficient vector $\beta_{3_{\mathrm{true}}}$ representing interaction effects is initialized as a sparse vector, where nonzero values are assigned to a subset of elements in the vector. In addition, a sparse coefficient vector $\beta_{2_{\mathrm{true}}}$ denoting the genetic main effects is also generated. The two vectors, $\beta_{2_{\mathrm{true}}}$ and $\beta_{3_{\mathrm{true}}}$, represent fixed effects that are subject to selection. Time-effect covariates $X_{\mathrm{it}}$ = $\left(1,t,t^{2}\right)$ are included to model longitudinal trends. The random effect design matrix $Z_{\mathrm{it}}$ includes both the intercept and slope terms. Random effects $\alpha_{i}$ are simulated for each subject to capture intra-subject correlation. According to model (2), the time-point-specific response $y_{it}$ is generated from the combined time effects, G×E main and interaction effects, and subject-specific random effects, with an additional error term independently drawn from a heavy-tailed distribution, specifically a t(2) distribution, in the following R example. All the variables that will be used later on as input for the mixedBayes package, including the response and covariates (genetic, environmental, and time-related), are required to be in a long format. The output includes the response vector y, covariates g, e and time effects X in a long format, and the true coefficients, stored as coef. The corresponding R code and its output are shown below. The code can also be accessed through the Figshare repository listed in the Data Availability Statement. 
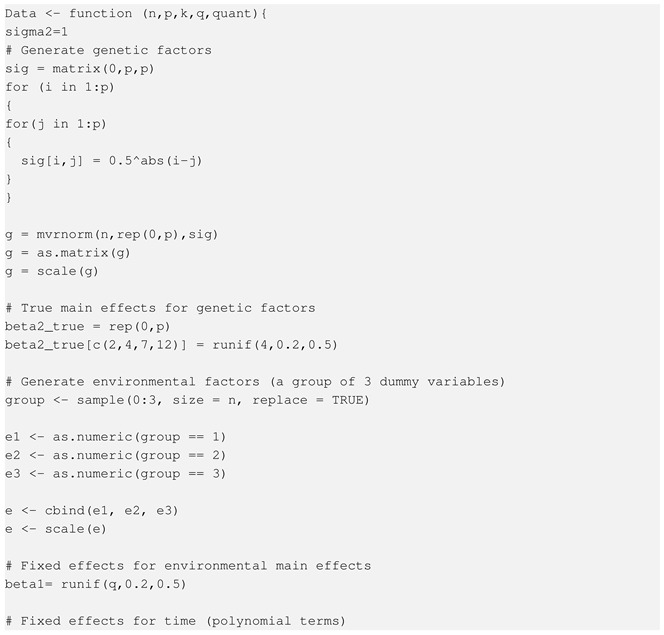
 
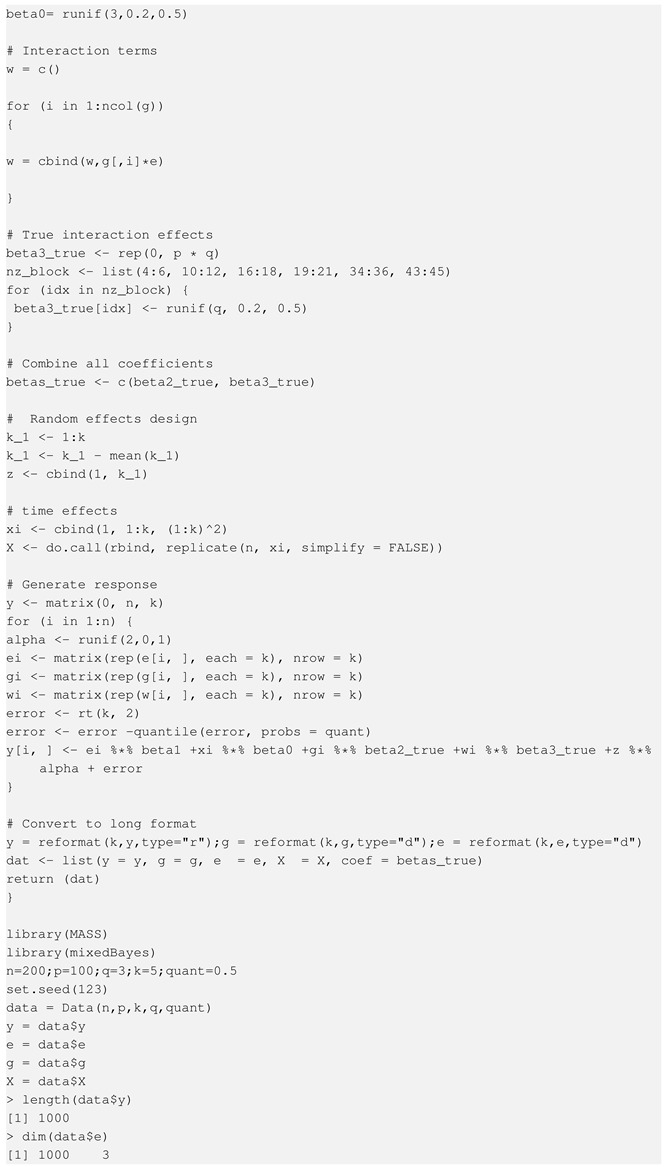
 
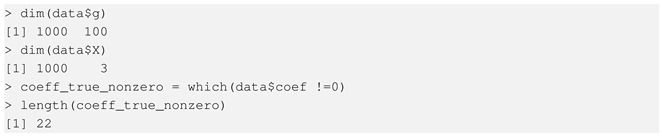


Moreover, the nonzero coefficients used in the data-generating process are stored in data$coef. By setting a random seed, the simulated dataset can be reproduced by re-running the Data function. With 100 genetic factors and 3 environmental variables, the model includes a total of 403 covariates representing main and interaction effects (excluding the intercept). Except for the 3 environmental main effects, all 400 genetic main effects and G×E interactions are subject to selection. We use the function mixedBayes to perform robust Bayesian bi-level variable selection at the 50% quantile level, imposing exact sparsity on the fixed effects via spike-and-slab priors. Posterior samples are collected via Gibbs samplers with 10,000 MCMC iterations, of which the first 5000 are treated as burn-ins, and Bayesian estimates are computed using posterior medians. The corresponding R code is as follows: 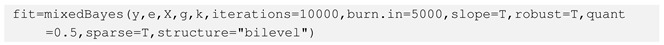


Since the true active predictors are known from the data-generating mechanism, their indices (index) can be obtained from the true coefficient vector (coeff). Identification performance is then evaluated by comparing the selected predictors with the true active set. Specifically, the number of true positives (TPs) is defined as the number of correctly identified active predictors, while the number of false positives (FPs) corresponds to the number of inactive predictors that are incorrectly selected. There are 4 main effects and 18 interaction effects, for a total of 22 true effects. The implementation is illustrated in the following code:
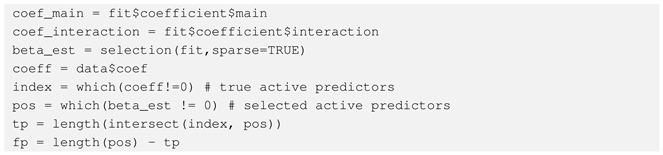


With sparse = TRUE, the selection() function identifies selected predictors under the median probability model by returning a binary vector beta_est consisting of 1’s and 0’s, where 1 indicates that the corresponding predictor is selected and 0 otherwise. When sparse = FALSE, selected predictors are determined using the 95% credible interval criterion. In this example, we use the median probability model since it better accommodates the two-group structure of spike-and-slab priors. The true positives (TPs) and false positives (FPs) are displayed in the R console. 19 out of 22 true effects are correctly identified. To facilitate comparison, the positions of the true and estimated nonzero regression coefficients—stored in the variables index and pos, respectively—are also presented. The first 20 elements of coef_main and coef_interaction as output from the R console are listed below. 
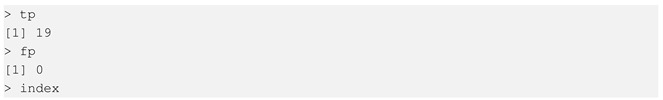
 
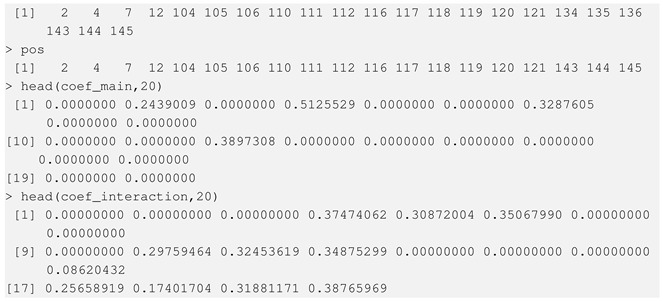


#### 4.1.2. Estimation

We provide details on how parameter estimation is evaluated. In Fan et al. (2025) [15], the accuracy of the regression coefficient estimates is examined in terms of TMAD, MAD, and NMAD. Specifically, the total mean absolute deviation (TMAD) is defined as

$$\mathrm{TMAD}=\frac{1}{M}\sum_{i=1}^{M}\frac{|{\hat{\beta_{2}}}^{\left(i\right)}-\beta_{2_{\mathrm{true}}}\left|+\right|{\hat{\beta_{3}}}^{\left(i\right)}-\beta_{3_{\mathrm{true}}}|}{p_{\beta_{2}\beta_{3}}},$$

where $p_{\beta_{2}\beta_{3}}$ denotes the total dimension of $\beta_{2}$ and $\beta_{3}$, ${\hat{\beta_{2}}}^{\left(i\right)}$ and ${\hat{\beta_{3}}}^{\left(i\right)}$ are the estimates obtained from the *i*th simulated dataset, and *M* is the total number of simulation replications. In our R example, *M* = 1, as we only show model fitting based on one replicate. In addition, we report the mean absolute deviation computed separately for coefficients that are truly nonzero (MAD) and for coefficients that are truly zero (NMAD), thereby allowing separate assessment of estimation accuracy for signal and noise components. The above estimation criteria are computed based on posterior median estimates obtained from the fitted model. In the following, we show R codes to calculate estimation errors in terms of TMAD, MAD, and NMAD based on one simulation replicate.
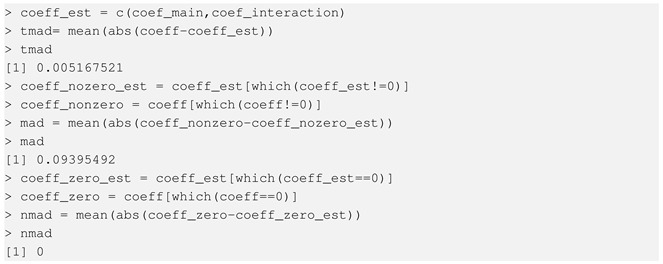


#### 4.1.3. Prediction

We use the function predict_mixedBayes from the R package mixedBayes to compute the prediction error in terms of the mean absolute error (MAE) across all subjects and observation times under model (2), where MAE is defined in Section 3.3. The R codes are shown below.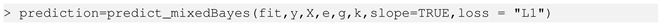 
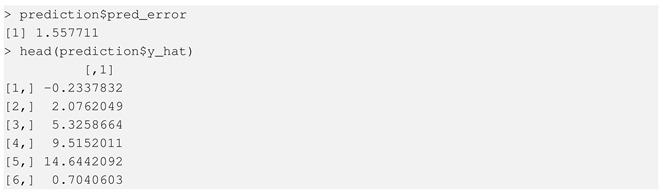


#### 4.1.4. Computational Time

Based on the R example in Section 4.1.1, the computational times under different combinations of sample size *n* and total dimensionality qp+p+1 are reported in Table 2, demonstrating that the *mixedBayes* package is computationally efficient.

#### 4.1.5. Convergence

**Figure 1:**
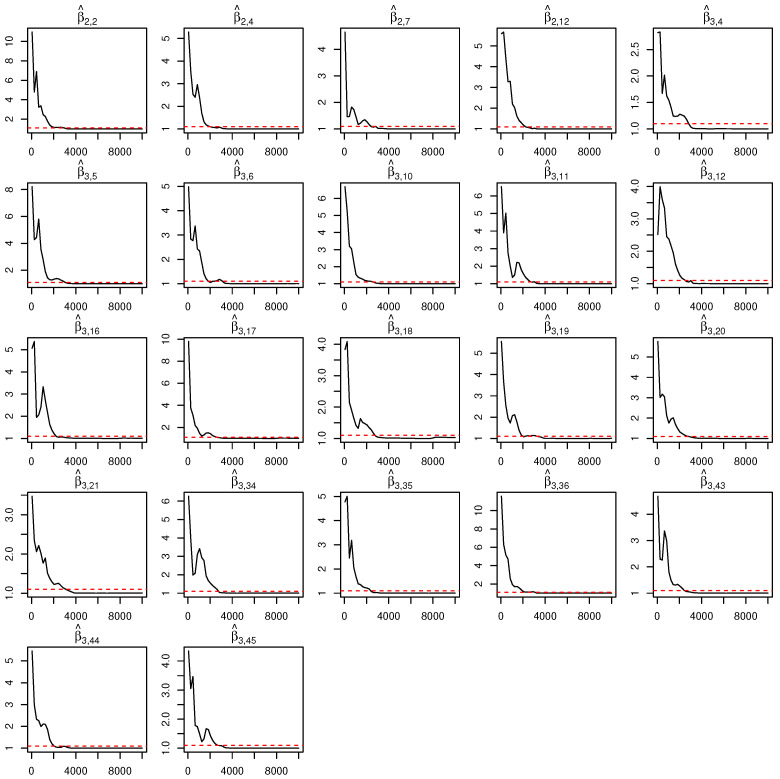
Potential scale reduction factor (PSRF) across iterations for nonzero coefficients (4 G main effects and 18 G×E interaction effects) in a simulation at the 50% quantile level under the model described in Section 4.1. Black line: The PSRF. Red line: The threshold of 1.1.

**Figure A1:**
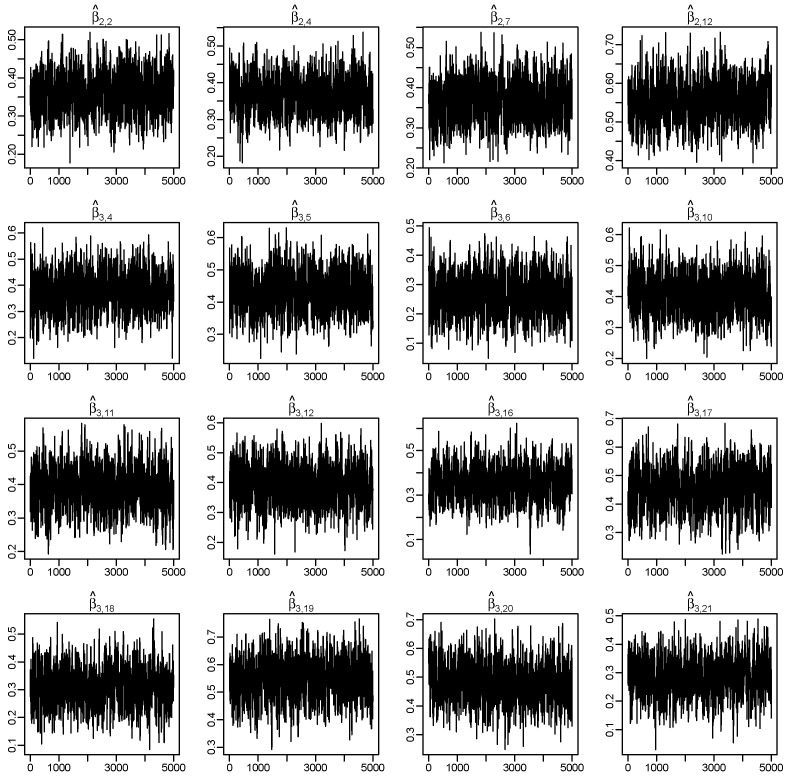
Trace plots for representative nonzero coefficients (4 G main effects and 12 G×E interaction effects) under the model described in Section 4.1 at the 50% quantile level after discarding the first 5000 iterations as burn-in.

We assessed MCMC convergence using the potential scale reduction factor (PSRF) [47,48]. Values of the PSRF approaching 1 indicate that Markov chains converge to stationary distributions. We adopted a threshold of 1.1 as the convergence criterion, as recommended by Gelman et al. (1995) [49]. PSRFs have been computed for all nonzero coefficients representing the 22 true main genetic and interaction effects. Figure 1 shows that all chains achieve satisfactory convergence after removal of the burn-in samples. In addition, trace plots are shown in Figure A1 in the Appendix A.

To further assess convergence and sampling efficiency, we have additionally evaluated the effective sample size (ESS) based on four parallel MCMC chains after discarding the first 5000 iterations as burn-in. The ESS is defined as $ESS=\frac{N}{1+2\sum_{k=1}^{\infty }\rho_{k}}$, where *N* denotes the number of post-burn-in samples, and $\rho_{k}$ is the lag-*k* autocorrelation. Larger ESS values indicate lower autocorrelation and more efficient posterior sampling. The ESS values have been computed using the coda package in R [50]. Table 3 summarizes the ESS values for nonzero coefficients, zero coefficients, and all coefficients for both main effects and interaction effects. Overall, the results indicate adequate mixing behavior and satisfactory sampling efficiency of the proposed MCMC algorithm. As expected in high-dimensional sparse Bayesian models, and as illustrated in Table 3, the ESS can vary substantially across coefficients, particularly for sparse parameters. Therefore, ESS should be interpreted with caution when assessing sampling efficiency in high-dimensional settings.

### 4.2. Identification via Bayesian FDR Under a Robust Linear Mixed Model

To further illustrate the Bayesian FDR-based selection procedure for mixed models [16], we consider a simulation example corresponding to model (3) introduced in Section 2.2. Rather than generating environmental factors (and thus, G×E interactions) at the group level, as in Section 4.1, we simulate the environmental variables from a multivariate normal distribution with an autoregressive covariance structure, $\sum_{ij}=0.5^{\left|i-j\right|}$. Consequently, the interaction effects no longer follow a grouped structure. In the following data-generating code, the sparse coefficient vectors $\gamma_{2\mathrm{true}}$ and $\gamma_{3\mathrm{true}}$, representing the main genetic effects and G×E interactions, are initialized as sparse regression coefficient vectors. Detailed R code is provided below and is also available at the Figshare repository listed in the Data Availability Statement section.
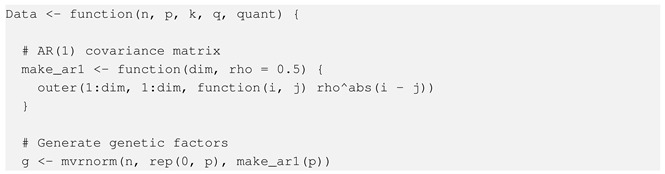

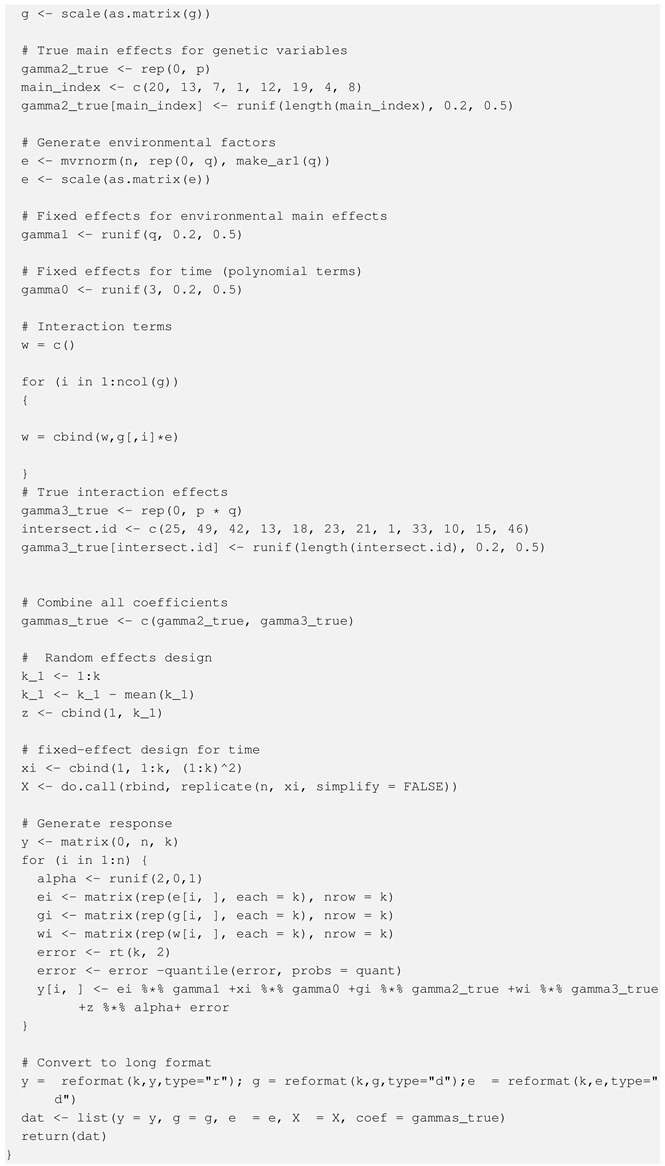
 
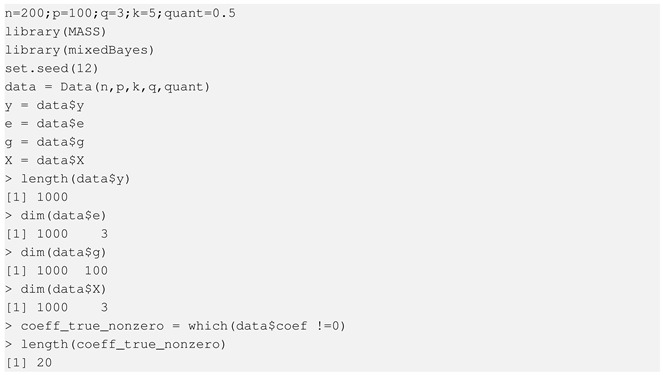


Of the 400 main and interaction effects under selection, only 8 genetic main effects and 12 interaction effects have nonzero coefficients. The numbers of true positives (TPs) and false positives (FPs) are computed based on the predictors, with posterior inclusion probabilities (PIPs) beyond the adaptive cutoff determined by the Bayesian false discovery rate (FDR) procedure defined in Equation (4). The adaptive threshold is determined according to a global FDR level fixed at 5% (i.e., c=0.05). R code for model fitting and variable selection is provided in the following R code: 
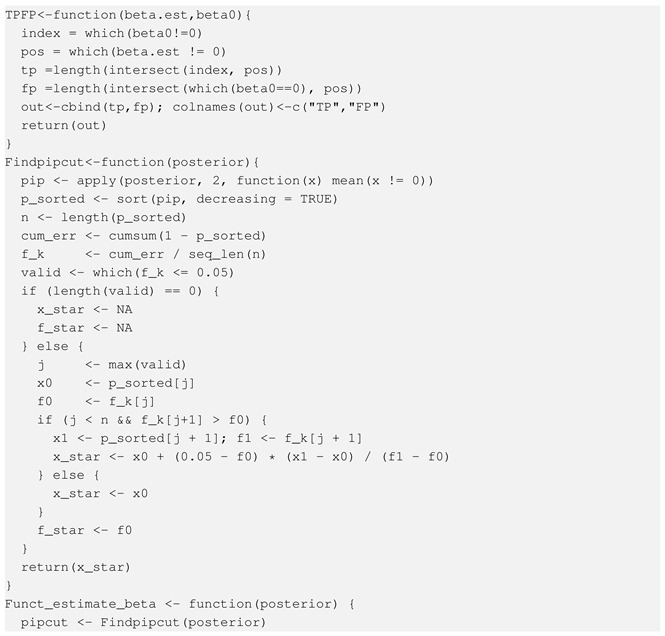
 
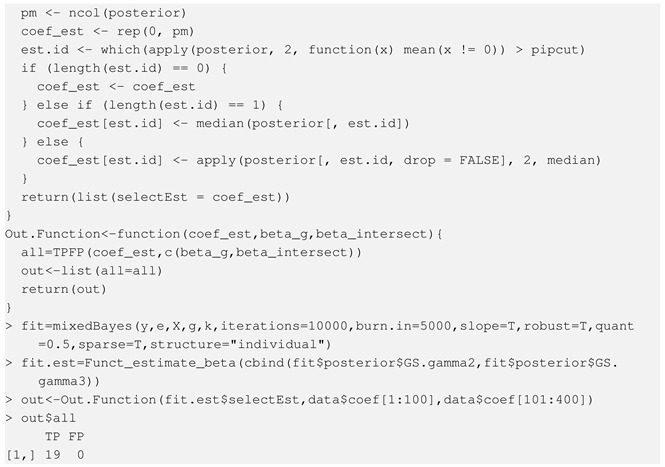


After fitting model (3) described in Section 2.2, posterior samples of $\gamma_{2}$ and $\gamma_{3}$ that correspond to genetic main effects and G×E interactions are extracted. For each coefficient, the posterior inclusion probability (PIP) is computed as the proportion of posterior samples in which the coefficient is nonzero. The function Findpipcut determines a PIP cutoff based on Bayesian FDR control at the 0.05 level. Specifically, variables are ranked according to their PIPs, and the cutoff is selected such that the estimated average false discovery rate among the selected variables does not exceed 5%.

The function Funct_estimate_beta then selects main and interaction effects whose PIPs exceed the Bayesian FDR cutoff. For selected effects, coefficient estimates are obtained using the posterior median, while non-selected coefficients are set to zero. Finally, the selected variables are compared with the true nonzero coefficients to evaluate variable selection performance in terms of true positives and false positives.

### 4.3. Remarks on R Examples

In both R examples presented in Section 4.1 and Section 4.2, the model errors are generated from a t(2) distribution. Compared with other commonly used heavy-tailed distributions, the t(2) distribution has infinite variance and, therefore, produces more extreme outliers. Comprehensive evaluations of model performance under Normal errors and other types of heavy-tailed model errors, including Laplace, contaminated Laplace mixtures, and skewed log-normal distributions, can be found in the numerical studies of Fan et al. (2025) [15] and Li et al. (2025) [16].

Currently, in both examples, the nonzero regression coefficients for important main and interaction effects are generated from Unif(0.2, 0.5). Users may explore different signal strengths, for example, stronger signals generated from Unif(0.4, 0.8), to further examine model performance. In addition, users may specify other types of model errors instead of t(2) to evaluate the robustness of the proposed methods under different error distributions. If interested, users can readily modify the above code to evaluate model performance averaged over multiple replications, along with corresponding standard deviations, under more challenging simulation settings.

## 5. Case Study

We utilized the package mixedBayes to analyze high-dimensional longitudinal data from the Childhood Asthma Management Program (CAMP) [51,52,53]. Access to the data may be requested through dbGaP using the accession number phs000166.v2.p1. In this study, children aged 5 to 12 years with a diagnosis of chronic asthma were enrolled in the study and followed for four years through scheduled visits. The outcome of interest is the forced expiratory volume in one second (FEV1), which measures the volume of air exhaled from the lungs within one second. We analyze longitudinal FEV1 measurements collected across 12 post-treatment visits under three treatment arms: budesonide, nedocromil, and placebo. In the original CAMP dataset, the treatment variable is encoded numerically, where trt = 4 corresponds to budesonide, trt = 8 corresponds to nedocromil, and trt = 9 corresponds to a placebo. In the repeated-measures one-way ANOVA design with the high-dimensional genetic factors introduced in Fan et al. (2025) [15], the treatment variable serves as the environmental factor and is encoded as a group of dummy variables. Specifically, trt_4 is defined as 1 for budesonide and 0 otherwise, and trt_8 is defined as 1 for nedocromil and 0 otherwise. Placebo is used as the baseline group. In addition, we include time effects through a design matrix X in the long format. Specifically, X consists of an intercept and the visit time (in months), where the first column is a vector of ones and the second column corresponds to the scheduled visit times. This matrix is constructed by repeating the visit times for each subject. With high-dimensional single-nucleotide polymorphisms (SNPs) as genetic factors, this study is exactly the longitudinal interaction study arising from the repeated-measure one-way ANOVA examined in Section 4.1. For demonstration purposes, we adopted the mixedBayes package to analyze a subset of 150 SNPs. Data formatting and model-fitting codes are provided below. 
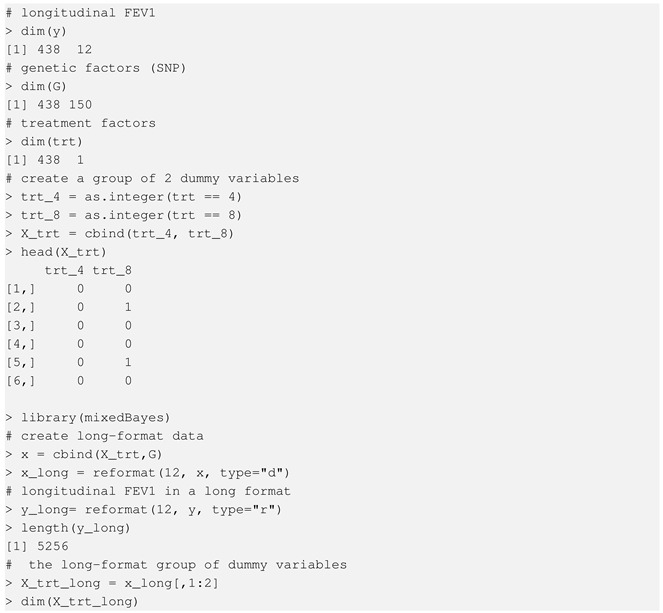
 
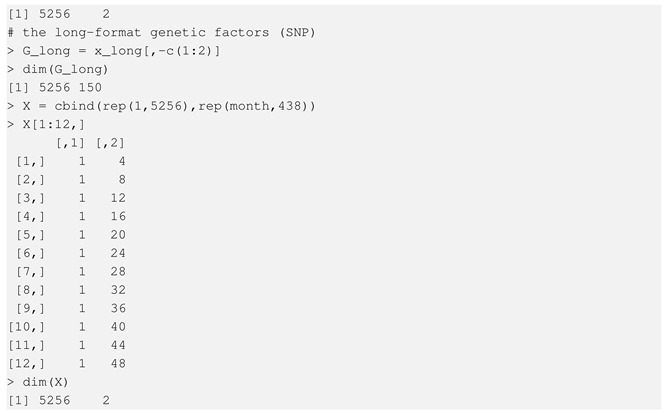


We have applied the robust Bayesian bi-level selection method under a random intercept-and-slope model to the real dataset using the mixedBayes function. 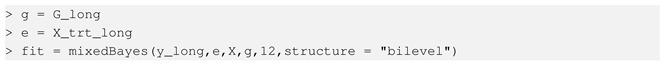


**Figure A2:**
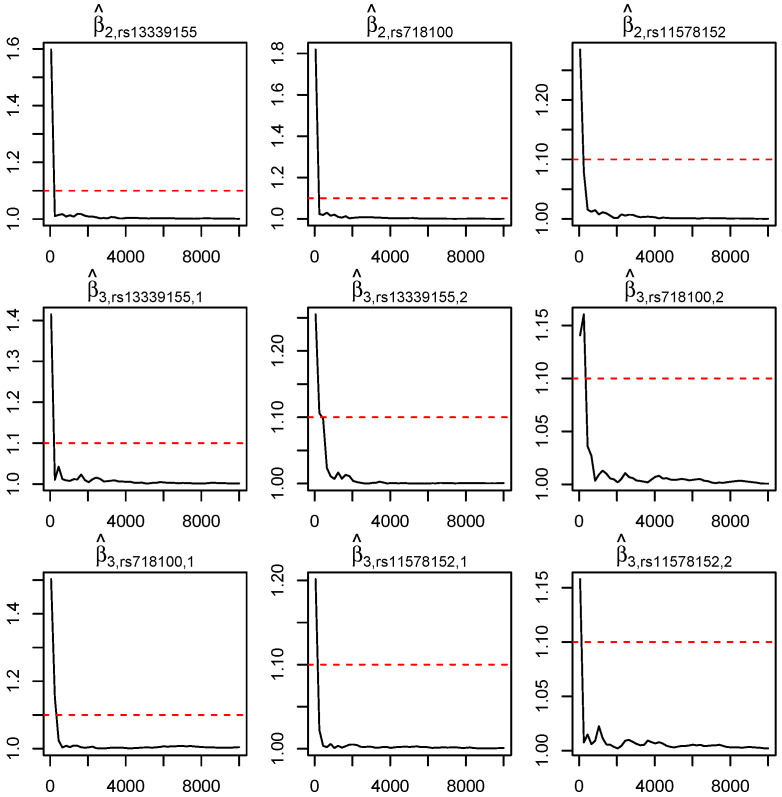
Potential scale reduction factor (PSRF) across iterations for selected SNPs (3 SNP main effects and 6 SNP×E interaction effects) from the case study at the 50% quantile level. Black line: The PSRF. Red line: The threshold of 1.1.

**Figure A3:**
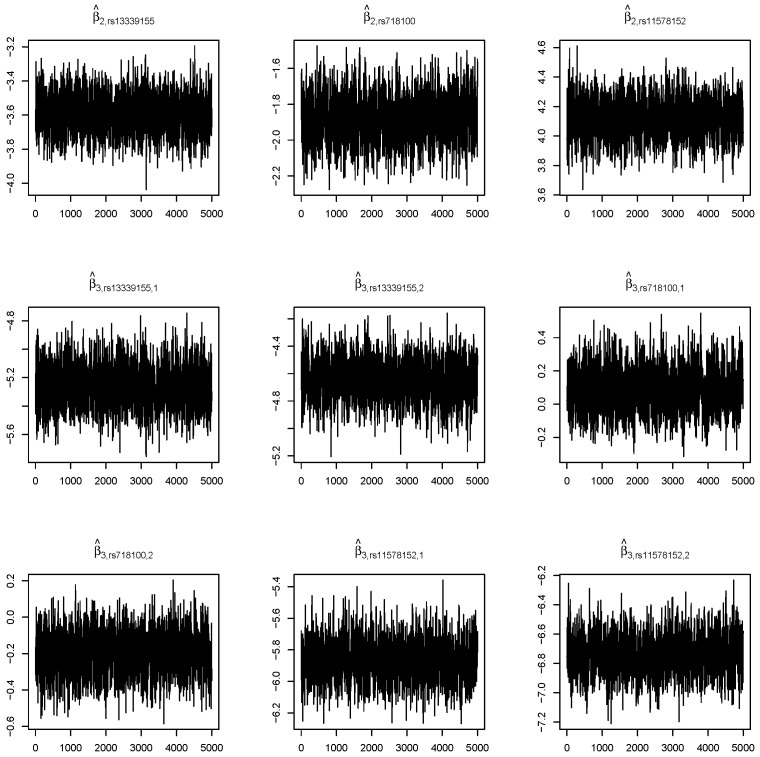
Trace plots for selected SNPs (3 SNP main effects and 6 SNP×E interaction effects) in the case study at the 50% quantile level after discarding the first 5000 iterations as burn-in.

The mixedBayes function provides estimates for the intercept, time-related covariates, treatment indicators, genetic effects, and G×E interaction terms. We provide the selected genetic main effects and G×E interactions in Table 4. Specifically, the first column lists the identified SNPs, while the remaining columns report the estimated coefficients for the SNP main effects and their corresponding interactions with the treatment factors. Model convergence diagnostics in terms of PSRF plots and trace plots are shown in Figure A2 and Figure A3 in the Appendix A, respectively.

Among the selected SNPs, rs718100, located in the *ILVBL* gene region, has previously been investigated in aspirin-exacerbated respiratory disease (AERD) among asthma patients [54]. In addition, rs13339155 is located near the *WWOX* gene region on chromosome 16q23.1. Previous studies have suggested that *WWOX* may play an important role in tumor suppression and lung cancer development [55]. Although further biological validation is needed, these findings suggest that the proposed method is capable of identifying potentially meaningful genetic markers and G×E interactions in longitudinal asthma studies.

## 6. Discussion

In this paper, we introduce the R package mixedBayes, which implements the longitudinal gene–environment interaction analyses proposed in Fan et al. (2025) [15] and Li et al. (2025) [16]. Our limited software review, together with Table 1, indicates that while numerous R packages have been developed for variable selection and for low-dimensional longitudinal analysis separately (under both frequentist and Bayesian frameworks), relatively few can address these two tasks simultaneously. In particular, due to the challenges of robust inference in high-dimensional settings [14], mixedBayes is among the few R packages that enable robust statistical inference for high-dimensional longitudinal studies.

Nevertheless, mixedBayes has certain limitations. It currently assumes that all subjects have the same number of repeated measurements and, therefore, cannot accommodate irregular observation schedules or missing data. Such limitations are partially due to the fact that the CAMP data analyzed in this paper, as well as the longitudinal lipid data analyzed in Fan et al. (2025) [15] and Li et al. (2025) [16], contain the same number of repeated measurements for all subjects and do not involve irregular or missing observations. The *mixedBayes* package can be readily extended to accommodate these characteristics when such real-world data become available to the authors. Moreover, only the response variable is measured longitudinally, while the genetic and environmental factors are assumed to be time-invariant. Future updates will aim to address these limitations. In addition, from a methodological perspective, the current version of mixedBayes focuses on linear G×E interactions, whereas substantial work has explored nonlinear interactions in cross-sectional settings [3]. Extensions to nonlinear gene–environment interactions are available in the R package *Blend* (version 0.1.2), which is publicly available on CRAN at https://CRAN.R-project.org/package=Blend (accessed on 1 May 2026). Furthermore, Bayesian inference may also be conducted using approximate Bayesian computation methods rather than MCMC, such as Wang et al. (2023) [56], to quantify the uncertainty of important main and interaction effects.

## Figures and Tables

**Table 2:** Computational time based on the R example in Section 4.1 at the 50% quantile. *p*: number of genetic factors. Time: CPU time (in seconds) for 10,000 MCMC iterations. The total number of regression coefficients to be estimated is qp+p+1, where *q* is the number of environmental factors. In this study, q=3.

*n* = 200		*n* = 500		*n* = 800	
** p=100 **	6.312	** p=400 **	55.912	** p=600 **	144.246
** p=200 **	10.968	** p=500 **	66.852	** p=800 **	177.652
** p=300 **	15.704	** p=600 **	79.193	** p=1000 **	416.022

**Table 3:** Convergence diagnostics for the model described in Section 4.1 based on four parallel MCMC chains. Effective sample size (ESS) is reported as mean (SD) for nonzero, zero, and all coefficients after discarding the first 5000 iterations as burn-in.

Parameter Group	Nonzero Coefficients	Zero Coefficients	All Coefficients
Main effects	1089.07 (888.10)	9175.08 (4426.94)	8851.64 (4622.33)
Interaction effects	1982.02 (724.67)	2417.19 (3059.52)	2391.08 (2972.83)

**Table 4:** Estimated regression coefficients for the identified SNP main effects and SNP-by-treatment interactions under the model described in Section 4.1.

SNP	SNP Main Effect	Budesonide × SNP	Nedocromil × SNP
rs11578152	4.209	−5.709	−6.800
rs7155089	–	0.126	−0.066
rs7630628	–	0.303	−0.127
rs992379	–	0.307	−0.052
rs1667540	0.061	–	–
rs10403708	–	0.044	0.106
rs243693	–	0.152	−0.160
rs17591211	4.206	−5.956	−6.952
rs6849562	–	−0.604	−0.101
rs925834	–	0.059	0.241
rs13339155	−3.695	−5.242	−4.840
rs1360367	0.075	–	–
rs447600	0.064	–	–
rs1539388	0.092	0.308	0.106
rs1359358	6.196	1.131	6.052
rs7630692	–	0.327	0.081
rs6505413	0.089	–	–
rs12890352	–	0.365	−0.016
rs7340644	−0.077	–	–
rs867322	–	0.165	−0.041
rs9522168	0.108	0.632	0.018
rs12149130	0.170	–	–
rs718100	−1.706	0.306	−0.238
rs4853602	0.000	0.442	0.161
rs10247061	0.157	0.529	0.346
rs3765306	0.077	0.407	0.118
rs777355	–	0.284	−0.088
rs1478971	1.748	–	–
rs6968039	0.084	–	–
rs12548055	–	0.326	−0.079
rs6575421	–	1.033	−0.072
rs10846561	0.030	–	–
rs7571528	6.276	1.829	6.190
rs287884	0.029	0.413	−0.151
rs11128375	0.015	–	–
rs10111289	–	0.016	−0.107
rs12708825	0.053	–	–
rs7828652	3.787	5.918	5.012

SNPs are included if either their main effects or corresponding treatment interaction effects are selected via the median probability model criterion, consistent with the weak hierarchy principle of the fitted model. Estimates are rounded to three decimal places; “–” indicates that the corresponding main or interaction effect is not selected.

## Data Availability

The data analyzed in the case study require authorized access and, therefore, cannot be publicly shared or included in the manuscript. Access requests should be submitted to dbGaP (accession number phs000166.v2.p1) at https://www.ncbi.nlm.nih.gov/projects/gap/cgi-bin/study.cgi?study_id=phs000166.v2.p1 (accessed on 23 April 2026). The R code examples presented in Section 4.1 and Section 4.2 are also publicly available at https://doi.org/10.6084/m9.figshare.32395461 (accessed on 25 May 2026).

## Abbreviations

ANOVA	Analysis of Variance
ALD	Asymmetric Laplace distribution
CAMP	Childhood Asthma Management Program
CRAN	Comprehensive R Archive Network
ESS	Effective sample size
FDR	False discovery rate
FP	False positive
GEE	Generalized estimating
GLMM	Generalized linear mixed model
LASSO	Least absolute shrinkage and selection operator
MAD	Mean absolute deviation
MAE	Mean absolute error
MCMC	Markov chain Monte Carlo
MPM	Median probability model
MSE	Mean squared error
PIP	Posterior inclusion probability
PGEE	Penalized generalized estimating
PSRF	Potential scale reduction factor
TMAD	Total mean absolute deviation
TP	True positive
QIF	Quadratic Inference Function
SNP	Single-nucleotide polymorphism
